# Barrier-Oriented Design of Next-Generation Polymeric Nanocarriers for Targeted Drug Delivery

**DOI:** 10.3390/molecules31111817

**Published:** 2026-05-25

**Authors:** Subin Lee, Yerim Kim, Jeongeun Kim, Kwang Suk Lim, Hyun-Ouk Kim

**Affiliations:** 1 Division of Chemical Engineering and Bioengineering, College of Art, Culture and Engineering, Kangwon National University, Chuncheon-si 24341, Gangwon-do, Republic of Korea; isubin0903@kangwon.ac.kr (S.L.); 03erinkim@kangwon.ac.kr (Y.K.); 202112527@kangwon.ac.kr (J.K.); 2 Department of Smart Health Science and Technology, Kangwon National University, Chuncheon-si 24341, Gangwon-do, Republic of Korea; 3 Institute of Fermentation of Brewing, Kangwon National University, Chuncheon-si 24341, Gangwon-do, Republic of Korea

**Keywords:** biological barriers, polymeric nanocarriers, stimuli-responsive polymers, surface engineering, targeted drug delivery

## Abstract

Targeted drug delivery remains difficult because multiple biological barriers interfere with the stable transport of therapeutics to the site of action. Polymeric nanocarriers have gained broad attention as delivery platforms since their composition and surface properties can be adjusted to improve circulation behavior and cellular delivery. This review discusses the major biological barriers involved in targeted drug delivery and describes how polymeric nanocarriers are engineered to overcome them. Major carrier types, including polymeric nanoparticles and micelles, are considered with emphasis on their physicochemical and interfacial features. Particular attention is given to surface engineering and stimuli-responsive design as key strategies for barrier transport and controlled cargo release. The review also highlights representative applications in anticancer, gene, protein, and vaccine delivery, together with translational issues such as biocompatibility, stability, reproducibility, scale-up, and regulatory acceptance.

## 1. Introduction

Targeted drug delivery has been widely investigated as a strategy to enhance therapeutic efficacy while minimizing toxicity in normal tissues [1,2,3]. Nevertheless, its clinical translation remains constrained by the many biological barriers that interfere with the movement of therapeutic agents from the administration site to the final site of action [4,5]. After administration, drugs must preserve their stability in biological fluids, avoid premature clearance, accumulate in diseased tissues, penetrate local biological environments, enter target cells, and, in many cases, undergo controlled intracellular release before meaningful therapeutic activity can be achieved [6,7,8,9,10]. This continuous transmission process is closely linked to the LADME process that leads to the release, absorption, distribution, metabolism, and excretion of drugs [11]. In the release stage, the polymer nanocarrier does not merely support the drug, but controls the timing and site of drug release through the design of polymer composition, degradable bonds, internal structures, and stimulation reactivity [12]. In the absorption stage, the drug is protected in the oral, mucous, and lung administration processes and helps it pass through the mucous layer or epithelial barrier [13,14,15]. In the distribution stage, blood circulation, immune evasion, organ distribution, and lesion tissue accumulation are adjusted through particle size, surface charge, hydrophilic coating, ligand presentation, and biomimetic surface modification [16]. In the metabolic stage, unstable therapeutic substances such as nucleic acids, proteins, peptides, and vaccine antigens are protected from enzymatic degradation [17,18]. In the excretion stage, the biodegradability of macromolecules, particle size, surface properties, and the safety of decomposition products determine the possibility of renal and hepatic clearance and long-term retention [19,20]. Therefore, the LADME process is an indicator of pharmacokinetics that explains at which stage and how polymer nanocarriers control the in vivo behavior of drugs, providing an important conceptual basis for organizing biological barrier-centered design strategies. These demands are especially critical for fragile and biologically active cargos such as nucleic acids, proteins, peptides, and vaccine components, because their therapeutic function depends not only on delivery efficiency but also on maintaining molecular integrity throughout transport [21,22]. For this reason, the field has advanced beyond simple encapsulation approaches toward nanocarrier systems that can be engineered to interact with biological environments in a more controlled and adaptive way [23,24,25].

Among the currently available delivery platforms, polymeric nanocarriers are particularly attractive because they provide extensive tunability at both the molecular and supramolecular levels [26,27,28]. Their properties can be modulated through polymer composition, molecular weight, block sequence, structural architecture, degradable linkage design, interfacial chemistry, and responsive functional groups [29,30,31]. This design flexibility allows regulation of drug loading, colloidal stability, circulation behavior, tissue transport, cellular uptake, intracellular trafficking, and triggered cargo release [32,33,34]. Accordingly, polymeric nanoparticles, micelles, polymersomes, dendrimers, nanogels, and hybrid biomimetic carriers have emerged as important systems for drug, gene, protein, and vaccine delivery [35,36,37]. Importantly, the biological performance of these carriers is not determined simply by particle formation itself, but by how precisely polymer chemistry and physicochemical properties are matched to the dominant transport barriers present in a given therapeutic setting [38,39,40].

**Scheme 1:**
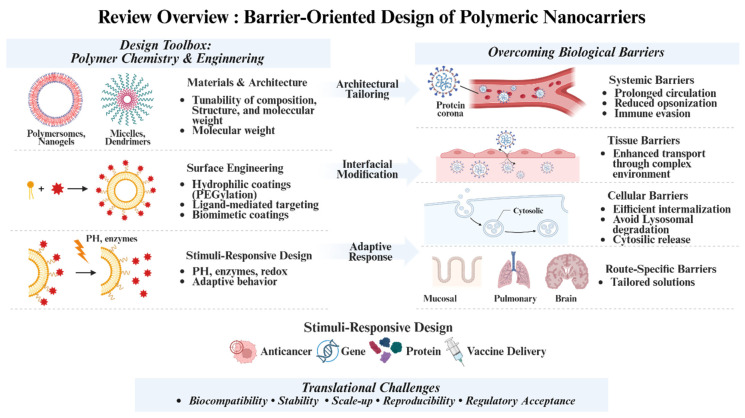
A schematic diagram illustrates the design strategies of polymer-based nanoplatforms for overcoming biological barriers in drug delivery. Engineering polymer structures, surface engineering, and stimuli-responsive properties of polymers enable effective drug delivery. Created with BioRender.com.

Recent progress in this field has increasingly shifted the focus from platform-centered development to barrier-informed design of polymeric nanocarriers. In this perspective, the key issue is not only which carrier can encapsulate a therapeutic cargo, but which molecular design features are most effective for overcoming the major biological limitations associated with a particular disease, administration route, and intracellular target [41,42,43]. Accordingly, surface engineering, ligand-mediated targeting, biomimetic coating, and stimulus-responsive design have received growing attention as strategies to improve immune evasion, tissue penetration, intracellular delivery, and transport across restrictive interfaces such as mucosal barriers and the blood–brain barrier (BBB) [44,45,46]. In this review, as summarized in Scheme 1, we discuss the major biological barriers that limit targeted drug delivery, the principal classes and design principles of polymeric nanocarriers, and their therapeutic applications together with key translational challenges. By integrating barrier biology with polymer-based material design, this review aims to provide a chemically grounded perspective on the rational development of next-generation polymeric nanocarriers for targeted drug delivery.

### Literature Search Strategy

The literature for this review was identified through systematic searches of PubMed, Web of Science, and Scopus, conducted between 2000 and 2025, with emphasis placed on studies published after 2020 to reflect current advances in the field. Search terms included “polymeric nanocarriers,” “drug delivery,” “biological barriers,” “nanoparticle design,” “targeted delivery,” “polymersomes,” “dendrimers,” “nanogels,” “stimuli-responsive,” “tumor penetration,” “endosomal escape,” and “blood–brain barrier,” applied individually and in combination. Original research articles, clinical and preclinical studies, and authoritative review articles reporting experimental data were considered for inclusion. Studies were excluded if they did not address polymeric carrier systems specifically, lacked sufficient mechanistic or experimental detail, or were not available in English. Reference lists of selected articles were also reviewed to identify additional relevant sources.

## 2. Biological Barriers in Targeted Drug Delivery

### 2.1. Systemic and Vascular Barriers

**Figure 1:**
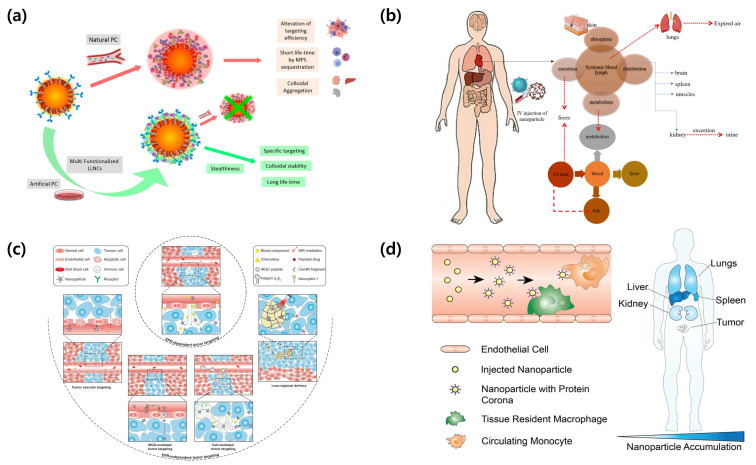
Schematic overview of nanoparticle design, biological barriers, and in vivo transport in drug delivery. (**a**) Preparation and functionalization of nanoparticles, including ligand conjugation, drug encapsulation, and stimuli-responsive release. Reproduced from Navarro-Marchal et al., 2023 [50], CC BY 4.0. (**b**) Systemic and vascular barriers after intravenous administration, including circulation stability, protein corona formation, immune clearance, and organ distribution. Reproduced from Haripriyaa and Suthindhiran, 2023 [51], CC BY 4.0. (**c**) Interactions with the biological microenvironment, including endothelial transport, tissue penetration, and extracellular matrix barriers. Solid arrows indicate the directional transport of nanoparticles across the endothelial layer into surrounding tissue, while dashed arrows represent diffusion-mediated or indirect transport pathways within the extracellular matrix. Reproduced from Fan et al., 2023 [52], CC BY 4.0. (**d**) Biodistribution and accumulation in major organs (lungs, liver, kidney, spleen, and tumor) and interactions with immune cells. Reproduced from Kim et al., 2025 [53], CC BY 4.0.

After intravenous injection, polymeric nanocarriers first face systemic and vascular barriers that determine two key outcomes, circulation stability and biodistribution. In blood, rapid interactions with plasma proteins, immune factors, and shear stress can alter colloidal stability and redefine the biological identity of the carrier [47]. Protein adsorption may also induce biomolecular corona formation, which can mask targeting ligands and accelerate clearance by the mononuclear phagocyte system, particularly in the liver and spleen [48]. In addition, endothelial barriers, uneven perfusion, abnormal vessel structure, and high interstitial pressure in diseased tissues further restrict extravasation and cause uneven tissue distribution [49]. Figure 1 presents these processes as the first major barrier stage in delivery. Therefore, effective nanocarrier design should consider two major factors, prolonged circulation and favorable early transport behavior, which are governed by surface hydrophilicity, charge, mechanical adaptability, and vascular interactions.

### 2.2. Tissue and Cellular Barriers

After crossing the vascular interface, nanocarriers must penetrate the local tissue environment and subsequently achieve productive intracellular delivery, yet both steps remain inefficient in many pathological conditions [54,55,56,57]. Tissue transport is frequently restricted by dense extracellular matrix structures, stromal cell barriers, irregular interstitial architecture, and limited diffusion, especially in solid tumors, fibrotic tissues, and inflamed lesions. Even when nanocarriers reach target cells, therapeutic success is not assured because surface association does not necessarily lead to effective internalization, and internalized cargos may remain trapped within endosomal compartments or be degraded in lysosomes before reaching their functional intracellular site. For this reason, delivery efficiency depends on a coordinated combination of carrier properties such as size, morphology, surface chemistry, ligand display, and responsive behavior under biological conditions [58,59]. These tissue and cellular barriers indicate that polymeric nanocarriers should be designed not only to improve tissue penetration but also to regulate intracellular trafficking and preserve cargo activity during release. This view highlights that material design must be closely matched to the transport requirements and intracellular fate of each therapeutic cargo.

### 2.3. Route-Specific Barriers in Oral, Mucosal, Pulmonary, and Brain Delivery

Beyond the barriers associated with systemic administration, each delivery route presents distinct biological obstacles and therefore demands route-specific nanocarrier design. In oral delivery, nanocarriers must withstand gastric acidity, digestive enzymes, bile components, mucus retention, and limited epithelial permeability before sufficient absorption can occur [60,61]. In mucosal delivery, local administration offers practical advantages, but rapid clearance and restricted transepithelial transport remain major limitations [62]. In pulmonary delivery, therapeutic performance is influenced by deposition behavior in the respiratory tract, interactions with mucus and surfactant layers, clearance by alveolar macrophages, and the sensitivity of the pulmonary epithelium to foreign materials [63]. Brain delivery is even more restrictive because the blood–brain barrier strongly limits transport through tight endothelial junctions, low transcellular permeability, and active efflux systems [64]. These route-dependent differences make it clear that a single polymeric nanocarrier design cannot be expected to perform optimally across all administration pathways. Accordingly, advanced carrier systems should be tailored to the specific biological demands of each route, and their utility should be evaluated according to how effectively their material composition and interfacial properties address the dominant transport barriers of that delivery setting.

## 3. Polymeric Nanocarriers and Design Principles

### 3.1. Classification and Major Material Types

Polymeric nanocarriers can be broadly classified by their structure, assembly behavior, and material composition, including micelles, polymersomes, nanogels, dendritic carriers, solid polymer nanoparticles, and hybrid systems combined with lipids, inorganic materials, or biomimetic membranes [24,26]. Each class offers a different balance of cargo loading, structural stability, release behavior, and biological interaction, making material selection a key factor in targeted drug delivery. Amphiphilic block copolymers are especially important because they readily form self-assembled nanostructures with tunable core-shell organization and can carry both hydrophobic and hydrophilic cargos [65]. In addition, biodegradable polyesters, polycarbonates, polysaccharides, and synthetic cationic polymers provide broader options for controlling degradation, surface properties, and cargo compatibility [30,66]. As polymer platforms continue to expand, carrier selection increasingly depends on the nature of the cargo, the delivery route, and the biological barrier to be overcome. Thus, classification should reflect the basic functional strengths and limitations that each material type introduces into the delivery system.

### 3.2. Key Physicochemical Properties Governing Delivery Performance

**Figure 2:**
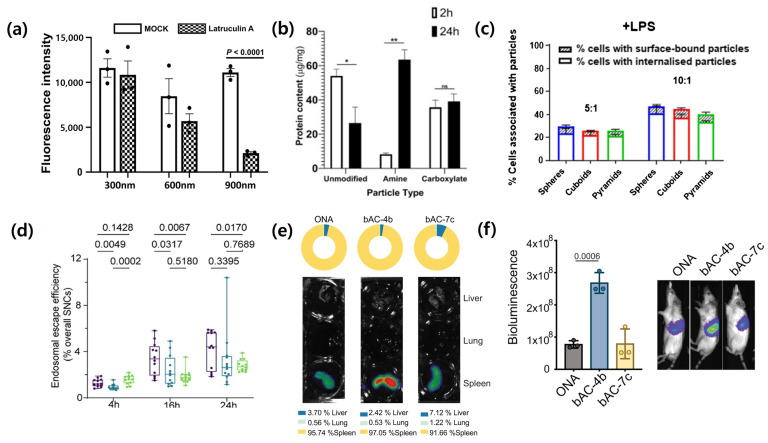
Influence of key physicochemical properties on the biological performance of polymeric nanocarriers. (**a**) Size-dependent pulmonary uptake of poly(lactic-co-glycolic acid) nanoparticles following intranasal administration. Reproduced from Baek et al., 2024 [67], licensed under CC BY 4.0. (**b**) Effect of protein corona formation on nanoparticle behavior depending on isolation and incubation conditions. White and black bars represent 2 h and 24 h incubation conditions, respectively. Statistical significance is indicated as follows: *, *p* < 0.05; **, *p* < 0.01; ns, not significant. Reproduced from Daramy et al., 2024 [69], licensed under CC BY 4.0. (**c**) Shape-dependent cellular uptake of nanoparticles, highlighting differences in internalization efficiency among geometries. Reproduced from Bruce et al., 2024 [70], licensed under CC BY 4.0. (**d**) Impact of nanoparticle stiffness on endosomal escape efficiency and intracellular signaling pathways. Reproduced from Zhang et al., 2025 [71], licensed under CC BY 4.0. (**e**) Role of surface charge in enhancing mRNA delivery and in vivo organ tropism. Reproduced from Li et al., 2023 [72], licensed under CC BY 4.0. (**f**) In vivo biodistribution and delivery efficiency of nanoparticles evaluated by bioluminescence imaging, with quantitative analysis of signal intensity across major organs. Reproduced from Li et al., 2023 [72], licensed under CC BY 4.0.

The biological performance of polymeric nanocarriers is shaped by physicochemical properties that interact with one another and with the biological environment in ways that produce markedly different delivery outcomes. Particle size is among the most consistently influential variables. Intranasally administered PLGA nanoparticles of three sizes displayed strongly size-dependent pulmonary phagocytic uptake in vivo, with biodistribution patterns differing substantially across particle sizes and administration routes [67] (Figure 2). Surface charge governs a distinct but equally important set of outcomes. Using bioluminescence imaging that charge modulation in nanoparticle formulations directly determines in vivo mRNA delivery efficiency and organ tropism, with quantitatively distinct biodistribution profiles observed across major organs. Particle shape and mechanical stiffness further modulate intracellular fate in ways that are not captured by size or charge alone. Cuboid-shaped particles achieved the highest macrophage cellular association (~25%) and uptake (~20%) compared to cylinders (~8%) and bar-shaped particles (~6%) [68], while softer nanocapsules exhibited superior cellular uptake but harder nanocapsules achieved approximately 1.8-fold greater endosomal escape efficiency through a ROS-mediated mechanism, revealing a direct trade-off between uptake and cytosolic delivery. Finally, protein corona formation can substantially alter the biological identity of a carrier regardless of its designed surface properties. Incubation conditions, shear flow, and isolation protocols all resulted in significant changes in the nanoparticle size, zeta potential, and adsorbed protein composition as a function of surface modification [69]. These results indicate that rational carrier design must treat size, charge, shape, stiffness, and surface composition as an integrated set of parameters rather than independent optimization targets.

### 3.3. Surface Engineering, Targeting, and Stimuli-Responsive Design

Surface engineering is a key design element in polymeric nanocarriers because the outer interface largely governs interactions with proteins, cells, and tissues under biological conditions. Strategies such as hydrophilic coating, ligand conjugation, charge shielding, and biomimetic surface modification can reduce nonspecific clearance, improve target recognition, and facilitate transport across biological barriers [10,73]. In parallel, active targeting based on receptor-binding ligands or cell-derived membrane components has been developed to promote site-selective delivery, although its practical value often depends on whether the carrier can first avoid systemic clearance and reach the target tissue [74]. Stimuli-responsive design further expands carrier function by allowing changes in structure, charge, or release behavior in response to local cues such as pH, redox conditions, enzymes, temperature, or external irradiation [75]. As a result, next-generation polymeric nanocarriers are increasingly designed as adaptive systems that respond dynamically to the biological environment rather than as passive drug reservoirs. This perspective is important because it directly links surface functionality and triggered behavior to improved selectivity, more effective intracellular delivery, and greater translational potential.

## 4. Major Polymeric Nanocarrier Platforms

### 4.1. Polymeric Nanoparticles and Micelles

Polymeric nanoparticles and micelles continue to be two of the most widely studied nanocarrier platforms because they combine formulation simplicity with broad relevance across diverse therapeutic cargos. Polymeric nanoparticles are commonly constructed from dense polymer matrices that support structural integrity, prolonged cargo retention, and tunable release behavior, making them suitable for many small-molecule drugs and, in some cases, biologics or imaging agents [76]. In contrast, polymeric micelles are usually assembled from amphiphilic block copolymers and are especially valuable for improving the solubility of poorly water-soluble compounds through their hydrophobic core and hydrophilic corona [77,78]. The properties of both platforms can be modulated by controlling polymer composition, molecular weight, block ratio, internal organization, and degradation profile, which allows their use to be adapted to different pathological settings and delivery routes [79]. However, their performance under physiological conditions is not without limitations, as premature cargo loss, micellar destabilization after dilution, and inconsistent in vivo retention remain important concerns. For this reason, polymeric nanoparticles and micelles should be regarded not only as fundamental delivery systems but also as reference platforms for determining whether more advanced polymeric nanocarriers offer a genuine biological advantage.

### 4.2. Polymersomes, Dendrimers, and Nanogels

Polymersomes, dendrimers, and nanogels are structurally more specialized polymer nanocarriers, allowing more precise control of cargo loading, spatial composition, and interaction with biological systems. Polymersomes are vesicular nanostructures typically formed from amphiphilic block copolymers, and their main advantage lies in their ability to encapsulate hydrophilic cargos within the aqueous core while accommodating hydrophobic cargos in the membrane domain at the same time. Relative to many lipid vesicles, they generally show higher membrane stability and more tunable permeability and degradation profiles. This dual-compartment capacity has been demonstrated preclinically: VP-DOX-PLs, prepared by loading verteporfin into the hydrophobic membrane and doxorubicin into the aqueous core of PLA-PEG polymersomes, achieved significantly superior antitumor efficacy compared to either sonodynamic therapy or chemotherapy alone in a xenograft mouse model, with selective drug accumulation at the tumor site confirmed by in vivo biodistribution analysis [80]. Dendrimer is a high-molecular monodisperse polymer with a clearly defined architecture characterized by multiple terminal functional groups, enabling accurate ligand attachment, multivalent bonding, and high-density cargo utilization. Despite these structural advantages, the complexity of synthesis associated with cationic surface charge and its clinical utility, including potential cytotoxicity, should be evaluated on a unique basis. VivaGel^®^ (SPL7013, astodrimer), a fourth-generation polylysine coordinated dendrimer that acts as a naphthalenedisulfonic acid-functionalized surface, exerts therapeutic effects through non-antibacterial biological mechanisms, preventing bacterial adherence to epithelial cells, and disrupting Gardnerella vaginalis-associated biofilms, a major cause of bacterial vaginosis (BV). Phase 3 clinical trials (NCT01577238) showed statistically significant clinical healing rates when 7 days of astodrimer gel vaginal administration was compared to placebo. The compound subsequently achieved key endpoints in two additional Phase 3 trials evaluating the prevention of recurrent BV (NCT02236156, NCT02237950), and the treatment was regulatory approved in Europe. These results represent one of the most advanced clinical translations performed by Dendrima-based nanocarriers to date [81]. Nanogels provide clear advantages through hydrated three-dimensional polymer networks, soft mechanical properties, swelling or degradation behavior in response to stimuli. These properties are particularly suitable for the delivery of proteins, peptides, and nucleic acids, which require compatibility of water and gentle release conditions. In an HT-29 xenograft mouse model, metronomic administration of DOX-loaded POx nanogels achieved significant tumor proliferation inhibition and long-term survival when compared with free DOX at the same dose, tumor-first accumulation by EPR effect was performed, and there were no adverse effects attributed to unload carriers [82]. Table 1 summarizes how these carrier platforms extend the structural diversity of polymeric nanocarriers, but its practical significance ultimately depends on whether the advancement of this architecture can yield clear biological benefits in the relevant deployment settings.

### 4.3. Hybrid and Biomimetic Polymer-Based Systems

Hybrid and biomimetic polymer-based systems have become important advanced carrier platforms because they combine synthetic polymers with complementary functional elements to address the limitations of conventional nanocarriers. Hybrid systems are typically built by combining synthetic polymers with additional functional components such as lipids, inorganic nanoparticles, peptides, imaging agents, or catalytic materials [106]. Through this design, they can achieve better structural stability, broader functionality, and more controlled responses to biological conditions or external stimuli. Biomimetic systems develop this concept further by incorporating features derived from natural systems, including cell membrane coatings, extracellular vesicle-like properties, and biologically active surface motifs [107]. These features can improve immune evasion, extend circulation time, and strengthen target recognition. Such systems are attractive because they combine the adaptable properties of synthetic polymer materials with selected advantages found in biological structures. However, this increased structural and compositional complexity also creates major challenges in reproducibility, batch-to-batch consistency, mechanistic understanding, large-scale production, and regulatory evaluation. Therefore, hybrid and biomimetic polymer-based systems should be considered highly promising but technically demanding platforms, and their translational value will depend not only on functional performance but also on whether they can be standardized and manufactured with sufficient reliability.

## 5. Polymeric Nanocarriers for Overcoming Biological Barriers

### 5.1. Evasion of Immune Clearance and Prolonged Circulation

A fundamental requirement for effective targeted drug delivery is the ability of polymeric nanocarriers to avoid rapid immune recognition and remain in circulation long enough to reach diseased tissues [108]. After entering the bloodstream, nanocarriers are immediately exposed to plasma proteins and phagocytic cells, both of which can promote opsonization and accelerate clearance by the mononuclear phagocyte system [109]. To reduce this early loss, polymeric carriers have been engineered with hydrophilic surface layers, charge moderated interfaces, low fouling chemistries, and biomimetic coatings that suppress nonspecific protein adsorption and decrease premature sequestration in the liver and spleen. In addition, particle size, surface composition, and structural stability must be carefully controlled to preserve colloidal integrity and delivery competence under physiological conditions [110]. These design strategies show that prolonged circulation is not achieved simply by masking the carrier from immune surveillance, but by establishing a balanced biointerface that minimizes premature clearance while preserving the ability to later interact with target tissues and cells. This distinction is important because excessive shielding may improve blood persistence but can also reduce vascular interaction, cellular association, and ultimately delivery efficiency at the target site.

### 5.2. Improved Tissue Accumulation and Penetration

**Figure 3:**
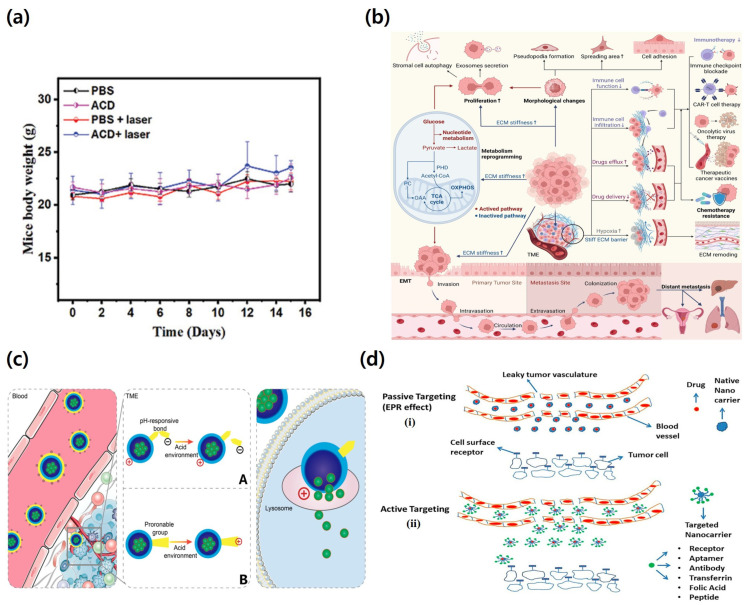
Strategies and Mechanisms for Enhancing Tissue Accumulation and Tumor Penetration. (**a**) Experimental timeline of photothermal therapy and recorded body weight of ACD-treated mice. Reproduced from Seo, Gyeongsu et al. (2026), [111], licensed under CC BY 3.0. (**b**) Metabolic reprogramming, ECM stiffening, immune modulation, and hypoxia in the tumor microenvironment (TME). Directional arrows indicate ECM stiffness-driven morphological changes, immune infiltration, drug efflux, and metastatic dissemination. Dashed arrows denote exosome-mediated intercellular communication. Colored symbols represent distinct cellular and molecular components as defined in the original figure legend. Reproduced from Mai et al. (2024), [112], licensed under CC BY 4.0. (**c**) Accumulation of pH-responsive nanocarriers in the TME followed by acid-triggered activation. Solid arrows indicate sequential steps of protonation or bond cleavage under acidic conditions and subsequent intracellular drug release. Reproduced from Liang et al. (2024), [113], licensed under CC BY 4.0. (**d**) Passive and active tumor accumulation strategies. Solid arrows indicate vascular extravasation via the EPR effect; dashed arrows denote receptor-mediated active targeting through ligands including antibodies, aptamers, transferrin, folic acid, and peptides. Reproduced from Seo, Gyeongsu et al. (2026), [111], licensed under CC BY 3.0.

Successful delivery requires more than survival in circulation, because polymeric nanocarriers must also accumulate at the target site and distribute beyond the immediate perivascular region into surrounding tissues. In many pathological environments, however, this step remains highly restricted by abnormal vascular organization, poor perfusion, elevated interstitial pressure, and dense extracellular matrix structures that limit both extravasation and deep tissue transport. To address these constraints, polymeric nanocarriers have been designed with optimized size, deformability, matrix interactive properties, and environmentally responsive behavior that support movement through confined biological spaces after leaving the vasculature. In some systems, triggered disassembly, local charge conversion, or size transformation has been used to improve diffusion and retention within diseased tissues. As illustrated in Figure 3, improved tissue accumulation and penetration serve as the critical link between systemic delivery and effective cellular action. For this reason, evaluation of carrier performance should distinguish simple localization near blood vessels from meaningful intratissue distribution, because perivascular accumulation alone often provides limited therapeutic benefit.

### 5.3. Enhanced Cellular Uptake and Intracellular Delivery

Successful delivery requires more than arrival at the target tissue. Polymeric nanocarriers must also be taken up by target cells and release their cargo at an intracellular site where therapeutic function can be maintained. Cellular entry is influenced by several carrier properties, including size, shape, surface charge, ligand density, and membrane affinity. After internalization, the final outcome is further shaped by endosomal trafficking, lysosomal degradation, and the ability of the carrier to support cytosolic or organelle specific release [114,115].

To improve these steps, advanced polymeric nanocarriers are often designed with receptor targeting ligands, membrane interactive domains, pH responsive groups, proton buffering moieties, or degradable linkages that promote endosomal escape and controlled intracellular cargo release [116]. These design features are particularly important for nucleic acids, proteins, and other biologically active macromolecules, because their therapeutic effect depends on avoiding degradative intracellular compartments and reaching the correct subcellular destination. Therefore, increased cellular uptake alone should not be considered sufficient proof of effective delivery. A more appropriate evaluation should determine whether internalization is linked to productive intracellular trafficking, efficient cargo release, and preservation of biological activity.

### 5.4. Transport Across Mucosal and Blood Brain Barriers

Mucosal surfaces and the blood–brain barrier represent two of the most restrictive biological interfaces in drug delivery because they are specifically organized to exclude foreign materials while preserving tissue homeostasis [10,117]. In mucosal delivery, polymeric nanocarriers must navigate mucus entrapment, rapid clearance, and limited epithelial transport, which has led to the development of systems with mucus penetrating surfaces, mucoadhesive balance, and localized responsive release behavior [118]. In brain delivery, the blood–brain barrier imposes additional limitations through tight endothelial junctions, low transcellular permeability, and active efflux transporters, requiring carriers to exploit receptor mediated transcytosis, adsorptive transport, or biomimetic interactions for meaningful access to the central nervous system. Polymeric nanocarriers offer important advantages here because their composition and interface can be precisely tuned for route-specific transport challenges while retaining cargo protection and release control [119]. These examples show that barrier specific carrier design is essential when the biological interface itself is the dominant determinant of delivery failure. They also support a broader discussion that future progress will depend on designing polymeric systems around route-specific transport biology rather than relying on one universally optimized platform.

### 5.5. Experimental Models for Evaluating Biological Barrier-Oriented Nanocarrier Design

As polymeric nanocarriers are increasingly designed according to specific biological barriers, their evaluation should also be performed using barrier-relevant experimental models. Immune clearance, tissue penetration, intracellular delivery, mucosal transport, and blood–brain barrier crossing involve distinct biological structures and transport mechanisms, so no single model can fully reproduce the complexity of in vivo delivery. Therefore, complementary in vitro and in vivo models are required to determine whether a given carrier design effectively addresses the intended biological barrier. In vitro models provide useful mechanistic information under controlled conditions, including carrier stability in biological fluids, immune recognition, tissue diffusion, cellular uptake, epithelial transport, and blood–brain barrier permeability. However, these simplified systems cannot fully reproduce dynamic blood flow, immune complexity, tissue heterogeneity, mucus turnover, or organ-level physiology. For this reason, in vivo pharmacokinetic, biodistribution, tumor-bearing, mucosal delivery, and brain delivery models remain necessary to confirm circulation behavior, tissue accumulation, barrier crossing, and therapeutic efficacy. Representative models, main evaluation endpoints, and relevant references are summarized in Table 2.

## 6. Therapeutic Applications and Translational Challenges

### 6.1. Anticancer, Gene, Protein, and Vaccine Delivery

**Figure 4:**
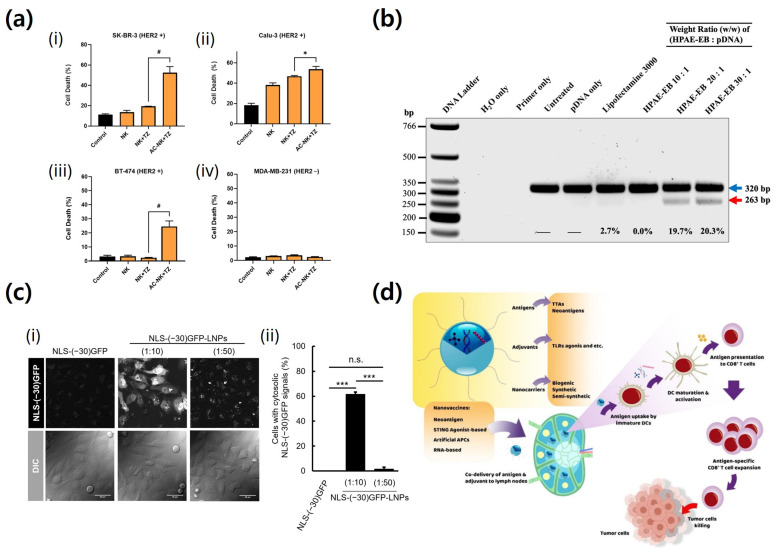
Strategic platforms for enhanced therapeutic delivery and anti-cancer efficacy. (**a**) In vitro anti-cancer efficacy of the antibody-capturing NK cell (AC-NK) surface engineering platform evaluated by cell death percentages across various cell lines, including (i) SK-BR-3, (**ii**) Calu-3, (**iii**) BT-474, and (**iv**) MDA-MB-231. To evaluate the combined effect with T-DM1 or SZ, HER2+ or Trop-2+ cancer cells were co-incubated with AC-NK cells, resulting in significantly enhanced apoptosis across all corresponding cell lines (**i**–**iv**) compared to controls. Results are expressed as mean ± SD (*n* = 3) with significance indicated by Student’s *t*-test (* *p* < 0.05, *# p* < 0.001). AC-NK: antibody-capturing NK cell. Reproduced from Lim et al. (2025), [133], licensed under CC BY 4.0. (**b**) CRISPR-C7 excision efficiency in HEK293 cells showed up to 20.3% editing via HPAE-EB (30:1 ratio), exceeding commercial controls. Significance. Reproduced from Kim et al. (2024), [84], licensed under CC BY 4.0. (**c**) Cytosolic delivery of NLS-(−30)GFP via DOP-DEDA-based LNPs. (**i**) Confocal laser scanning microscopy (CLSM) images displaying the intracellular distribution of NLS-(−30)GFP (2.5 μM) after 6 h treatment across various formulations. Scale bar: 50 μm. (**ii**) Quantitative analysis of cells showing cytosolic fluorescence. n.s., not significant; *** *p* < 0.001. Reproduced from Hirai et al. (2021), [134], licensed under CC BY 4.0. (**d**) Nanovaccines deliver antigens and adjuvants to lymphoid tissues, triggering DC-mediated CD8+ T cell activation. These T cells then infiltrate the tumor microenvironment to selectively eliminate cancer cells. Reproduced from Fang et al. (2022), [135], licensed under CC BY 4.0.

Polymeric nanocarriers are widely used in anticancer, gene, protein, and vaccine delivery because their properties can be tailored to different cargos [128]. In cancer therapy, they improve tumor accumulation, limit off-target exposure, and support controlled drug release [129]. In gene delivery, they protect nucleic acids and promote cellular uptake, endosomal escape, and cytosolic release [130]. Protein and peptide delivery is more difficult because these cargos are easily degraded and poorly transported across membranes [131,132]. Vaccine delivery adds further complexity because antigens must be protected and efficiently delivered to immune cells with proper intracellular processing. Figure 4 demonstrates that the value of polymeric nanocarriers depends not simply on cargo loading but on how well carrier design matches cargo-specific barriers, target tissue demands, and intracellular delivery requirements. Therefore, these systems should be assessed according to each therapeutic application rather than treated as interchangeable platforms.

### 6.2. Biocompatibility, Stability, Scale-Up, and Regulatory Issues

Although polymeric nanocarriers have progressed markedly in material design, their clinical translation is still restricted by unresolved issues in safety, batch consistency, scalable manufacturing, and regulatory evaluation [136]. Biocompatibility should not be judged only by short-term cytotoxicity, because immunogenicity, degradation products, long-term tissue exposure, and route-dependent tolerance also determine whether a system is clinically acceptable. In the clinical translation of the polymeric nanocarrier, not only the toxicity of the therapeutic substance, but also the removal of the delivery body itself and the possibility of accumulation in the organ must be considered. Biodegradable macromolecules may be decomposed into low molecules by decomposition bonds and then removed primarily by the kidney or hepatic biliary tract [137]. On the other hand, non-biodegradable or slow-degradation polymers are likely to accumulate in mononuclear phagocyte-related tissues such as the liver and spleen, and may increase the risk of chronic inflammation, tissue remodeling, and fibrosis if repeatedly administered [138]. Therefore, the safety evaluation of polymer-based nanocarriers should include not only short-term cytotoxicity, but also decomposition products, organ distribution, clearance pathways, and indicators of inflammation and fibrosis after repeated administration.

Stability is another key concern, since nanocarriers may aggregate, release cargo too early, degrade during storage, or undergo surface alteration after administration, all of which can reduce reproducibility and compromise performance [139]. In addition, successful scale-up requires manufacturing processes that preserve particle size, surface characteristics, cargo loading, and release behavior with minimal batch-to-batch variation under practical conditions. Regulatory assessment also becomes more demanding as carrier structure becomes more complex, requiring clear identification of composition, mechanism related attributes, critical quality parameters, and safety profiles. Taken together, these limitations show that clinical translation depends on linking laboratory level design more closely to manufacturable and well controlled product development. Accordingly, the future value of polymeric nanocarriers will depend not only on therapeutic efficacy, but also on reliable production, consistent quality, and regulatory acceptance.

## 7. Conclusions

**Figure 5:**
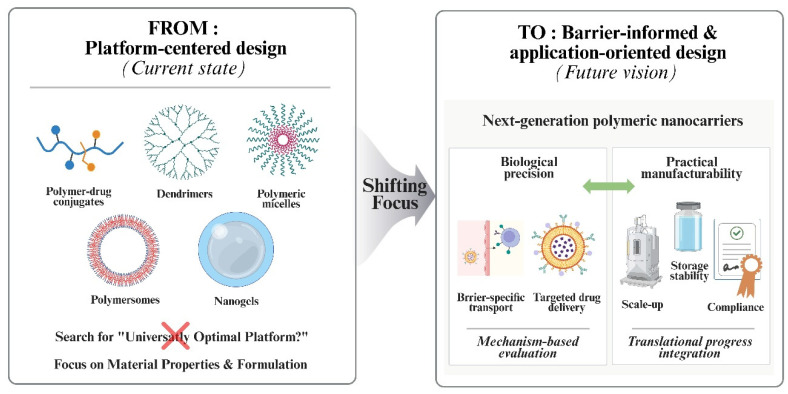
Paradigm shift from platform-centered to barrier-informed and application-oriented design of polymeric nanocarriers. No single platform is universally optimal across diverse clinical needs. Next-generation carrier design integrates disease-specific biological barriers, therapeutic objectives, and practical manufacturability to improve translational potential. Created with BioRender.com.

Polymeric nanocarriers have progressed well beyond their original role as simple drug carriers and are now recognized as multifunctional delivery systems that can be tailored to overcome the main biological barriers limiting targeted therapy. Their overall performance during circulation, tissue penetration, cellular uptake, intracellular trafficking, and route-specific transport is shaped by the combined effects of polymer chemistry, physicochemical properties, surface engineering, and stimulus-responsive functions. As outlined throughout this review, no single carrier platform is universally superior, because delivery success depends on how precisely each system is matched to the dominant biological barrier, the characteristics of the therapeutic cargo, and the demands of the administration route. In this regard, the most important advance in the field is not merely the growing diversity of carrier platforms, but the stronger ability to connect polymer design with barrier-specific delivery requirements (Figure 5).

Future progress should place less emphasis on platform expansion alone and more emphasis on mechanism-driven, application-focused design. Greater effort is needed to clarify how polymeric nanocarriers behave in complex in vivo settings, how barrier-overcoming capacity can be assessed with meaningful transport and functional metrics, and how therapeutic outcomes can be reproduced under clinically relevant conditions. At the same time, translational development should incorporate biocompatibility, storage stability, manufacturing consistency, and regulatory feasibility from the earliest design stages rather than treating them as secondary considerations after proof of concept. Accordingly, the next generation of polymeric nanocarriers will be defined not only by structural complexity, but also by their ability to unite biological accuracy with practical manufacturability. Continued progress will therefore depend on carrier systems that are mechanistically sound, clinically scalable, and reliably effective across diverse therapeutic settings.

## Figures and Tables

**Table 1:** Comparative design features of major polymer-based nanocarrier platforms for targeted drug delivery.

Platform	Structural Organization	Main Loading Mode	Cargos Most Suited	Delivery Advantages	Barriers Addressed	Main Limitations	Design Variables	Ref.
Polymeric nanoparticles	Dense solid colloidal matrix formed from biodegradable or non-biodegradable polymers	Encapsulation, adsorption, or matrix entrapment	Small molecules, hydrophobic drugs, some nucleic acids	High stability and sustained release	Circulation and distribution control	Limited macromolecule loading	Size and degradation rate	[83,84,85]
Polymeric micelles	Core-shell self-assemblies of amphiphilic block copolymers	Hydrophobic core solubilization, shell conjugation	Poorly water-soluble drugs, some imaging agents	Enhanced solubility and simple formulation	Aqueous solubility and penetration	Dilution induced dissociation	Block ratio and CMC	[86,87,88]
Polymersomes	Vesicular block copolymer assemblies with aqueous core and polymeric bilayer	Hydrophilic loading in the core and hydrophobic loading in the membrane	Small molecules, proteins, peptides, nucleic acids, combination cargos	Dual compartment loading and membrane stability	Cargo protection and release control	Complex fabrication processes	Membrane thickness and ligands	[89,90,91]
Dendrimers	Highly branched monodisperse macromolecules with multivalent terminal groups	Surface conjugation, internal cavity loading, electrostatic complexation	Small molecules, nucleic acids, imaging probes, targeting ligands	Precise architecture and multivalent binding	Cellular uptake and targeting	Synthetic complexity and toxicity	Generation number and charge	[83,92,93]
Nanogels	Hydrated crosslinked polymer networks with soft nanoscale architecture	Network entrapment, electrostatic loading, affinity interactions	Proteins, peptides, nucleic acids, hydrophilic drugs	Water compatibility and stimulus response	Cargo degradation and endosomal escape	Low mechanical strength	Crosslinking density and swelling	[94,95,96]
Nanocapsules	Core-shell particles with liquid or semi-solid inner compartment surrounded by polymer shell	Reservoir loading in inner core, shell functionalization	Hydrophobic drugs, oils, combination therapeutics	Efficient loading and release modulation	Premature leakage and off-target exposure	Shell rupture risk	Shell thickness and stability	[93,97,98]
Polymer–drug conjugates	Soluble or particulate systems formed by covalent linkage between polymer and drug	Covalent conjugation through cleavable or non-cleavable linkers	Small molecules especially cytotoxic agents	Improved pharmacokinetics and reduced toxicity	Circulation stability and stoichiometry	Linker dependent activity	Linker chemistry and molecular weight	[83,99]
Polyplexes	Electrostatic complexes formed between cationic polymers and anionic nucleic acids	Ionic complexation	pDNA, siRNA, mRNA, antisense oligonucleotides	Nucleic acid condensation and protection	Cellular uptake and endosomal escape	Charge associated toxicity	N/P ratio and charge density	[84,100,101]
Polymer–lipid hybrids	Composite particles combining polymeric core or scaffold with lipid shell or interface	Matrix entrapment plus lipid-assisted loading	Small molecules, nucleic acids, proteins, co-delivery systems	Combined stability and biocompatibility	Enhanced uptake and circulation balance	High formulation complexity	Core shell ratio and composition	[93,102,103]
Biomimetic hybrids	Polymer carriers coated or hybridized with cell membrane, extracellular vesicle-like layer, or bioactive membrane motifs	Core loading with biomimetic surface presentation	Drugs, proteins, nucleic acids, immunomodulators	Enhanced immune evasion and targeting	Immune clearance and cell recognition	Reproducibility and quality control	Membrane type and coating quality	[91,104,105]

**Table 2:** Representative models for evaluating biological barrier-oriented polymeric nanocarriers.

Biological Barrier	Representative Models	Main Endpoints	Ref.
Immune clearance and circulation	Serum/protein corona assays; macrophage uptake; PK/biodistribution	Opsonization; blood half-life; liver/spleen accumulation	[120,121]
Tissue accumulation and penetration	3D tumor spheroids; ECM hydrogel/transwell models; tumor-bearing animals	Penetration depth; intratumoral distribution; tumor retention	[122,123]
Cellular uptake and intracellular delivery	Flow cytometry; confocal colocalization; functional cargo assays	Cellular uptake; endosomal escape; cargo activity	[124]
Mucosal barriers	Mucus diffusion assays; epithelial transwell models; mucosal delivery animals	Mucus penetration; epithelial transport; local retention	[125]
Blood–brain barrier	BBB transwell/co-culture models; BBB-on-chip; brain delivery animals	TEER/permeability; transcytosis; brain accumulation	[126,127]

## Data Availability

No new data were created or analyzed in this study. Data sharing is not applicable to this article.

## Abbreviations

The following abbreviations are used in this manuscript:

BBB	Blood–Brain Barrier
CMC	critical micelle concentration
pDNA	plasmid DNA
siRNA	small interfering RNA
mRNA	messenger RNA
ECM	extracellular matrix
EPR	enhanced permeability and retention
AC-NK	antibody-capturing NK cell
T-DM1	Trastuzumab emtansine
SZ	Sacituzumab
HER2	Human Epidermal Growth Factor Receptor 2
Trop-2+	Trophoblast cell surface antigen 2-positive
HEK293	Human Embryonic Kidney 293 cells
HPAE-EB	Hyperbranched poly(amino ester) containing endosomal buffering groups
LNP	lipid nanoparticle
CLSM	confocal laser scanning microscopy
DC	dendritic cell
